# The Journal of Appalachian Health: A Year of Growth and Impact

**DOI:** 10.13023/jah.0604.01

**Published:** 2025-01-29

**Authors:** Noah Wren, Bradley Firchow, Emily Wilson, Randy Wykoff

**Affiliations:** East Tennessee State University

**Keywords:** Appalachia, Appalachian health, public health, readership

## Abstract

The Journal of Appalachian Health continues to grow in strength and reach, serving as a critical platform for research and discussion on the unique health challenges and opportunities in Appalachia, as well as how the challenges and opportunities Appalachia faces are local manifestations of broader regional and global phenomena. Over the past year, we have witnessed a remarkable expansion in our readership, reflecting the increasing demand for evidence-based insights into the health of our region.

The *Journal of Appalachian Health* continues to grow in strength and reach, serving as a critical platform for research and discussion on the unique health challenges and opportunities in Appalachia. Over the past year, we have witnessed a remarkable expansion in our readership, reflecting the increasing demand for evidence-based insights into the health of our region as well as the relevance of our region’s experience to other parts of the country and, further, the world.

In 2024, total readership based on downloads from Digital Commons and PubMed reached 39,907 downloads, representing a significant increase over 2023’s readership of 24,181. This growth highlights *JAH’s* expanding influence and the value of our authors’ contributions. Notably, Digital Commons data alone indicates that our articles were downloaded in more than 147 different countries and 1,653 institutions.

This progress would not be possible without the dedication of our authors and peer reviewers, whose commitment to scholarly excellence ensures the *Journal of Appalachian Health’*s continued success. We also extend our deepest gratitude to the Appalachian Regional Commission for their steadfast support in advancing health equity and research dissemination across the region through their generous financial support. In an era where timely, evidence-based knowledge is essential for addressing global health challenges, open access scientific journals play a critical role in ensuring that research is freely available to the policymakers, practitioners, and communities who need it most.

As we look ahead, we remain committed to promoting high-quality research, amplifying diverse perspectives, and strengthening collaborations that drive positive health outcomes in Appalachia, across the country, and around the world. Thank you for being part of this journey and, especially, for allowing *JAH* to be a part of our mutual goal of improving the health and well-being of the people of Appalachia and beyond.

